# A library of functional recombinant *Plasmodium falciparum* merozoite surface proteins

**DOI:** 10.1186/1475-2875-9-S2-O8

**Published:** 2010-10-20

**Authors:** Cecile Crosnier, Madushi Wanaguru, Brian McDade, Julian Rayner, Gavin J Wright

**Affiliations:** 1 Cell Surface Signalling laboratory, Wellcome Trust Sanger Institute, Cambridge, CB10 1 HH, UK; 2 Sanger Malaria Program, Wellcome Trust Sanger Institute, Cambridge, CB10 1HH, UK

## 

Parasites of the *Plasmodium* genus are the etiological agents responsible for malaria, and *Plasmodium falciparum* is by far the most virulent in humans. All the clinical symptoms of malaria result from the invasion of human erythrocytes by the parasite's merozoites. Proteins displayed at the merozoite surface are believed to be critical for erythrocyte invasion and are therefore good vaccine candidates, yet in many cases their precise function remains poorly understood. One reason for this is the difficulty in producing large amounts of functional recombinant parasite proteins. Expressing natively-folded extracellular proteins in bacterial and cell-free systems is often difficult; therefore eukaryotic systems such as yeast and mammalian cells, in which exogenous extracellular proteins are directed towards the secretory pathway for correct folding, are often more suitable.

In this study, we have attempted to express the extracellular domain of 49 secreted or cell-surface merozoite proteins from *P. falciparum* (11 GPI-anchored, 12 peripheral, nine micronemal, 13 rhoptry, and four other proteins), using transient transfection of human embryonic kidney (HEK) 293E cells. To improve the production of functional recombinant proteins, all coding sequences were codon-optimised for expression in human cells and any potential N-linked glycosylation site modified so as to prevent inappropriate addition of large glycans that would not be present on native proteins. Endogenous signal sequences were replaced by an exoge¬nous mammalian signal sequence to promote correct addressing of the recombinant proteins to the secretory pathway. Using this approach, we were able to detect the expression of 39 proteins (80%) at their expected molecular weight by western blot including for example a full-length ectodomain of MSP1. Of the 10 proteins that gave no expression or unexpected sizes, seven were rhoptry proteins and the remaining three belonged to the GPI-anchored, micronemal and other categories.

Following purification, yields reached up to 1.4 mg of protein from a 50 mL transfection. Using the known MSP1-MSP7 and EBA175-GlycophorinA inter¬actions as examples, we showed that the recombinant proteins were able to specifically bind to their interaction partner, demonstrating that they were correctly folded and functional. We believe this resource will make a significant contribution to the functional analysis of merozoite proteins and may be useful as vaccine candidates.

